# A Case of Multilevel Aortic Disease Treated Using Cardiatis Multilayer Flow Modulator

**DOI:** 10.3400/avd.cr.21-00031

**Published:** 2021-09-25

**Authors:** Mafalda Massara, Antonino Alberti, Andrea Cutrupi, Vittorio Alberti, Gaetana Franco, Pietro Volpe

**Affiliations:** 1 Unit of Vascular and Endovascular Surgery, Grande Ospedale Metropolitano “Bianchi-Melacrino-Morelli,” Reggio Calabria, Italy; 2 Intensive Care Unit, Emergency-Urgency Department, Grande Ospedale Metropolitano “Bianchi-Melacrino-Morelli,” Reggio Calabria, Italy

**Keywords:** multilevel aortic disease, multilayer flow modulator endograft, spinal cord ischemia

## Abstract

A recent systematic review and meta-analysis shows that synchronous and metachronous thoracic and abdominal aortic aneurysms are present in 19.2% of cases. The management remains controversial: elective simultaneous TEVAR and EVAR could increase morbidity due to increased aortic coverage during a single procedure, longer operative times, increased blood loss, and greater contrast exposure. Conversely, simultaneous thoracic endovascular aortic repair (TEVAR) and endovascular aneurysms repair (EVAR) prevent the need for two interventions, reduces future access site complications, and obviates interval aortic complications.

We present a case of a multilevel aortic disease treated in three stages: EVAR, TEVAR, and exclusion of an increasing aortic visceral penetrating aortic ulcer through a multilayer flow modulator endograft with an optimal result.

## Introduction

Currently, the infrarenal abdominal aorta represents the most common site of aneurysm formation, followed by the thoracic aorta.^1)^

Based on a recent systematic review and meta-analysis,^2)^ approximately 19.2% of patients affected by an abdominal aortic aneurysm (AAA) have a synchronous or metachronous (SM) thoracic aortic aneurysm (TAA), and women present a two-fold higher than man this association of AAA and TAA.

The management of SM TAA and AAA has been discussed by several authors. Some authors claim that concomitant thoracic endovascular aortic repair (TEVAR) and endovascular aneurysms repair (EVAR) can be performed successfully with minimal morbidity and mortality rate for patients.^3)^

Other authors proposed a staged repair: simultaneous TEVAR and EVAR involve covering a long tract of the aorta, with subsequent increased risk of spinal cord ischemia due to perfusion interruption of collateral vessels such as intercostal, pelvic, and hypogastric arteries. Additionally, a more contrast medium is used, with potential contrast-induced nephrotoxicity and a longer operative time is required. Thus, a two-staged endovascular repair should prevent the potential contrast-induced nephropathy and the risk of spinal cord ischemic injury.^4)^

In the present paper, we report our experience with the management of SM descending thoracic aortic voluminous penetrating aortic ulcers (PAUs) and a symptomatic AAA in a 66-year-old man.

## Case Report

A 66-year-old man presented in the Emergency Room of our hospital complaining of the sudden onset of abdominal pain. He was a strong smoker and was affected by chronic obstructive pulmonary disease, hypertension, dyslipidemia, and ischemic heart disease previously treated through percutaneous transluminal coronary artery angioplasty and stenting.

**Figure 1:**
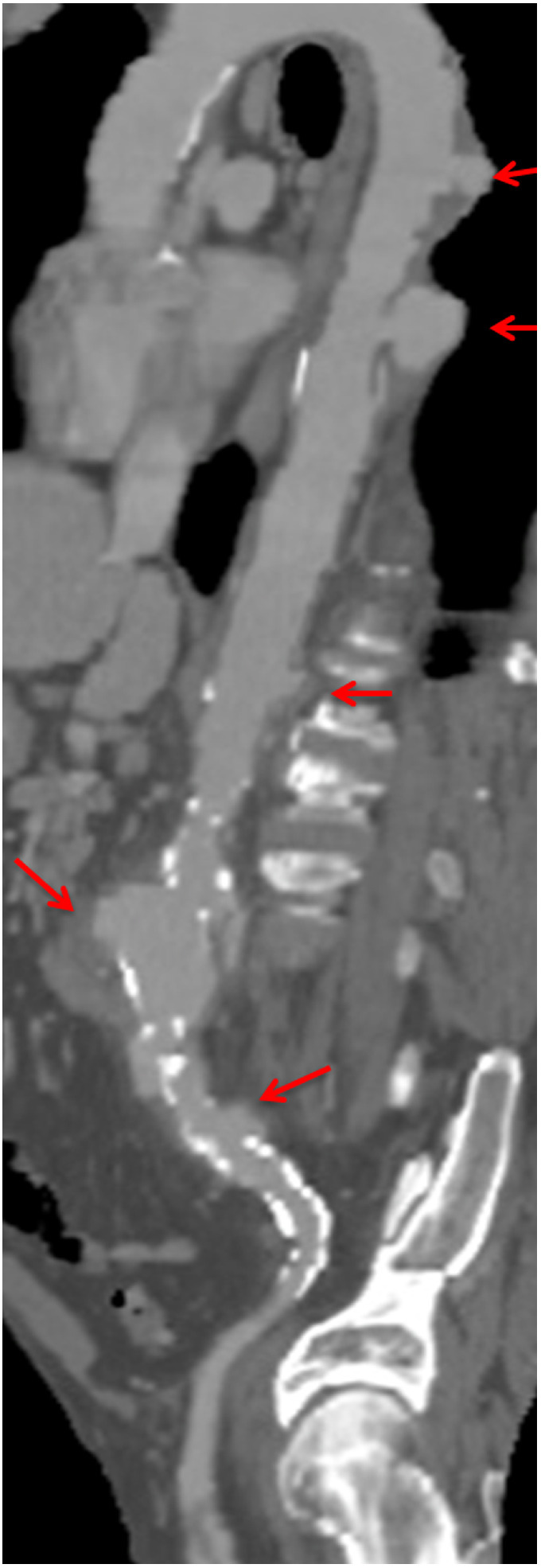
Fig. 1 Axial computed tomography images showing thoracic penetrating aortic ulcers, abdominal and left common iliac artery aneurysms, with chronic occlusion of the right iliac axis.

An urgent computed tomography (CT) scan revealed the concomitant presence of an infrarenal AAA with chronic asymptomatic right iliac axis occlusion and left common iliac artery aneurysm, two voluminous PAUs localized at the level of the descending thoracic aorta with a diameter of 41 and 45 mm and another posterior PAU at the level of visceral arteries origin (diameter of 30 mm) (**Fig. 1**).

After cardiological and anesthetic evaluation and a blood sample to exclude the infective nature of the aneurysms, we planned a multistage treatment for our patient excluding an elective fenestrated endovascular abdominal repair or a T-branch because he was symptomatic for abdominal pain and needed an urgent operation.

Thus, he was first submitted to EVAR in urgent time. The patient was operated on in the Hybrid Room of our hospital under local anesthesia, recurring to an aorto-uni-iliac stent graft (Endurant, Medtronic Vascular, Santa Rosa, CA, USA). The final angiography demonstrated the correct endoprosthesis deployment, in absence of endoleaks. After 3 days, the patient was discharged in good health.

Two months later, the patient returned to our Department and he underwent the planned TEVAR using a GORE® TAG® endoprosthesis (W.L. Gore and Associates, Flagstaff, AZ, USA) under epidural anesthesia, associated with a crossover prosthetic femoro–femoral bypass to reperfuse the right hypogastric artery, to reduce the risk of spinal cord ischemia. Because of the chronic right iliac axis occlusion, we recurred to a left percutaneous brachial artery access and to a left surgical common femoral artery access. To prevent spinal cord ischemia, we recurred to spinal fluid drainage, maintaining the drainage at 5 ml/h, with a spinal fluid pressure of 10 mmHg and a mean blood pressure of 90 mmHg. After the operation, the patient was monitored in the intensive care unit for 48 h. He did not develop paraplegia, so the spinal fluid drainage was removed, and he was discharged 3 days later in good health.

**Figure 2:**
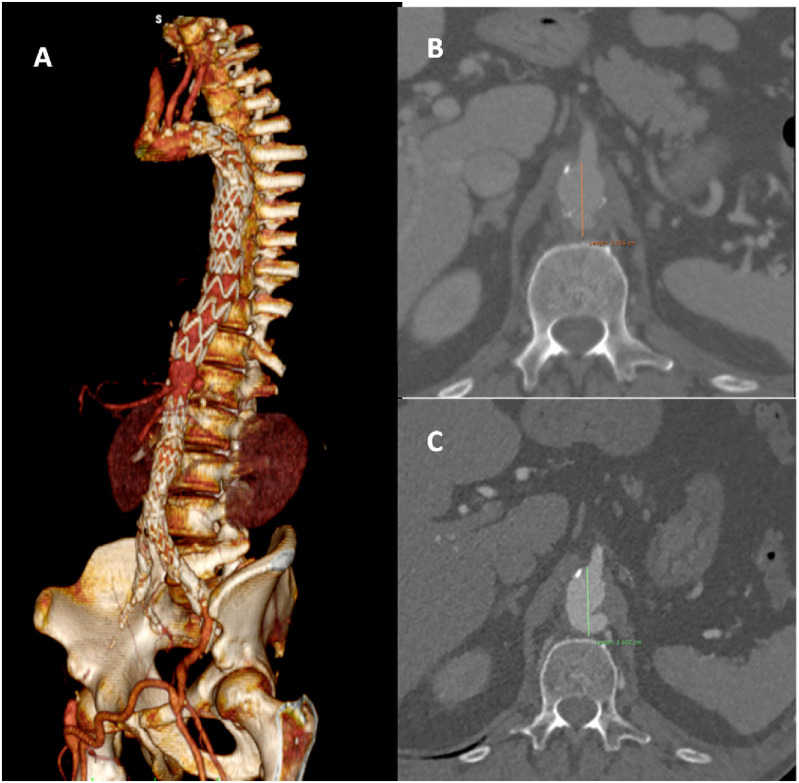
Fig. 2 Three-dimensional computed tomography (CT) reconstruction in lateral view showing the correct deployment of both thoracic and abdominal aorta, the femoro–femoral crossover bypass patency, and the residual posterior penetrating aortic ulcer (PAU) of the visceral aorta (**A**). Visceral aortic PAU of 30 mm at the first CT scan (**B**), increased to 36 mm at the last CT scan (**C**).

**Figure 3:**
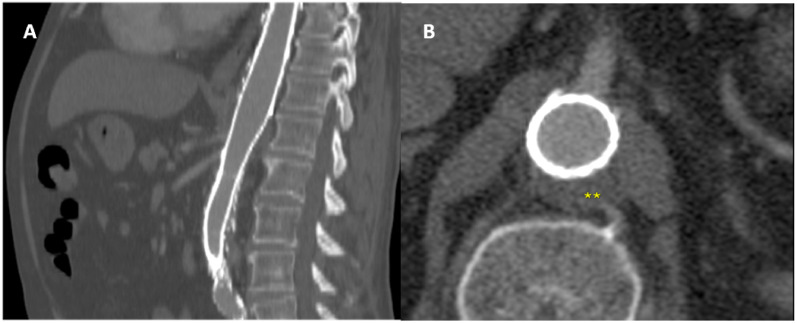
Fig. 3 Sagittal (**A**) and axial (**B**) computed tomography images showing the Cardiatis multilayer correctly deployed and the complete thromboses of the posterior visceral penetrating aortic ulcer.

The patient was submitted to a strict follow-up to exclude complications and to monitor the size of the remaining PAU at the level of the visceral aorta. Three months after the last operation, a CT scan control showed the correct deployment of both thoracic and abdominal endoprosthesis, the regular patency of the crossover femoro–femoral bypass (**Fig. 2A**), and the increase of the PAU size from 30 to 36 mm (**Figs. 2B** and **2C**). Thus, because of this very fast growth of the lesion, to prevent a sudden aortic rupture, the surgeon decided to cover this PAU using a multilayer stent (Cardiatis, Isne, Belgium), also able to maintain the visceral arteries patency and to prevent paraplegia, having already been covered a long tract of the aorta. The Cardiatis stent was deployed under local anesthesia through a percutaneous ultrasound-guided left femoral artery access just above the previous anastomosis between the common femoral artery and prosthesis, with a good final result. He was discharged 2 days after the operation, and a CT scan control at 1, 3, 6 and 12 months showed the regular patency and deployment of both the abdominal and thoracic endoprosthesis and of the multilayer stent, with complete exclusion and thromboses of the PAU already 1 month after Cardiatis deployment (**Figs. 3A** and **3B**).

## Discussion

The infrarenal abdominal aorta is the most common site of aortic aneurysm formation, followed by the thoracic aorta.^1)^ The prevalence of AAA is 9% in patients older than 65 years, and the incidence of TAA is at 5.9–10.4 patients per 100,000 person-years.^1–5)^

Regarding SM AAA and TAA, a recent review and meta-analysis^2)^ reported that 15.2% of men and 30.7% of women affected by AAA had SM TAA. Additionally, women suffering from AAA had a two-fold increased risk of having an SM TAA than men, and diabetes mellitus seems to be associated with a 43% decreased risk of having SM TAA.

Regarding the management of SM AAA and TAA, literature results are controversial.

Elective simultaneous TEVAR and EVAR treatment can increase the morbidity rate because of a longer operative time with subsequent increased blood loss and increased potential nephrotoxicity due to the use of a major quantity of contrast medium; additionally, the simultaneous coverage of long tracts of the aorta can increase the spinal cord ischemia rate. Conversely, simultaneous TEVAR and EVAR prevent the need for two interventions, can reduce future access site complications, and obviates interval aortic complications that may occur with a staged strategy.^4)^

In our patient, we planned a multistage endovascular repair comprising EVAR followed by TEVAR and crossover femoro–femoral prosthetic bypass, first, because of the sudden onset of abdominal pain that required an urgent EVAR without the possibility to plan a FEVAR or a T-branch endoprosthesis deployment and, second, to reduce the risk of spinal cord ischemia in a patient with chronic occlusion of the entire right iliac axis and requiring the coverage of the entire abdominal aorta and of a long part of the thoracic aorta. Additionally, the posterior PAU at the level of the visceral aorta increased in diameter over time, so at the last stage, we had to cover this lesion.

In our opinion, the better option to treat the increasing PAU at the level of the visceral aorta in a patient already submitted to TEVAR and EVAR was represented by a multilayer flow modulator (Cardiatis, Isnes, Belgium) endograft deployment.

This stent modulates a laminar flow within the stent and the aneurysm sac, guaranteeing patency of visceral collaterals arising from the covered aorta, whereas the thrombosis of the aneurysmal sac occurs over time, so in the immediate perioperative and postoperative periods, the aneurysmal sac is not promptly excluded from the blood pressure and the risk of rupture may still be present.^6,7)^

However, in our patient already submitted to TEVAR and EVAR, it represented the better choice achievable in a short time and in a minimally invasive way. In fact, this stent does not require customization, is available in a wide range of sizes, and can used also in the presence of a complex anatomy because it does not require cannulation of branch vessels and extensive measurements regarding visceral side branch location, diameters, and angulation.^6–9)^

However, despite the countless advantages, there remains controversy regarding its use because the aneurysm is not always effectively excluded for the first months after stent deployment.^6–10)^

## Conclusion

SM TAA and AAA occur in 19.2% of patients. The management of a multilevel aortic disease remains controversial. In our case, the presence of a symptomatic AAA led us to plan a multistage procedure with urgent EVAR followed by TEVAR and crossover femoro–femoral bypass, recurring to spinal fluid drainage to reduce the risk of spinal cord ischemia. The multilayer Cardiatis stent allowed us to exclude the increased posterior PAU of the visceral aorta in our patient, with the maintenance of visceral artery patency and thromboses of the PAU within 1 month from the operation, offering a good and fast solution in a patient already submitted to TEVAR and EVAR.
